# Integrated Single-Cell Bioinformatics Analysis Reveals Intrinsic and Extrinsic Biological Characteristics of Hematopoietic Stem Cell Aging

**DOI:** 10.3389/fgene.2021.745786

**Published:** 2021-10-19

**Authors:** Xiangjun Zeng, Xia Li, Mi Shao, Yulin Xu, Wei Shan, Cong Wei, Xiaoqing Li, Limengmeng Wang, Yongxian Hu, Yanmin Zhao, Pengxu Qian, He Huang

**Affiliations:** ^1^ Bone Marrow Transplantation Center, The First Affiliated Hospital, Zhejiang University School of Medicine, Hangzhou, China; ^2^ Institute of Hematology, Zhejiang University, Hangzhou, China; ^3^ Zhejiang Engineering Laboratory for Stem Cell and Immunotherapy, Hangzhou, China; ^4^ Zhejiang Laboratory for Systems and Precision Medicine, Zhejiang University Medical Center, Hangzhou, China; ^5^ Center of Stem Cell and Regenerative Medicine, Zhejiang University School of Medicine, Hangzhou, China

**Keywords:** hematopoietic stem cells, aging, single cell integrated analysis, cell cycle, inflammation

## Abstract

Hematopoietic stem cell (HSC) aging, which is accompanied by loss of self-renewal capacity, myeloid-biased differentiation and increased risks of hematopoietic malignancies, is an important focus in stem cell research. However, the mechanisms underlying HSC aging have not been fully elucidated. In the present study, we integrated 3 independent single-cell transcriptome datasets of HSCs together and identified Stat3 and Ifngr1 as two markers of apoptosis-biased and inflammatory aged HSCs. Besides, common differentially expressed genes (DEGs) between young and aged HSCs were identified and further validated by quantitative RT-PCR. Functional enrichment analysis revealed that these DEGs were predominantly involved in the cell cycle and the tumor necrosis factor (TNF) signaling pathway. We further found that the Skp2-induced signaling pathway (Skp2→Cip1→CycA/CDK2→DP-1) contributed to a rapid transition through G1 phase in aged HSCs. In addition, analysis of the extrinsic alterations on HSC aging revealed the increased expression levels of inflammatory genes in bone marrow microenvironment. Colony formation unit assays showed that inflammatory cytokines promoted cellular senescence and that blockade of inflammatory pathway markedly rejuvenated aged HSC functions and increased B cell output. Collectively, our study elucidated the biological characteristics of HSC aging, and the genes and pathways we identified could be potential biomarkers and targets for the identification and rejuvenation of aged HSCs.

## Introduction

Hematopoietic stem cell (HSC) aging is accompanied by reduced self-renewal ability, myeloid-biased differentiation, impaired homing capacity and other defects in hematopoietic reconstitution function (Liang et al., 2005; Rossi et al., 2005; Dykstra et al., 2011; Li et al., 2020). Emerging studies have elucidated the intrinsic and extrinsic mechanisms of HSC aging. For instance, microarray analysis of HSC expression profiles revealed that HSC aging was accompanied by downregulation of genes mediating lymphoid specification differentiation and upregulation of genes involved in specifying myeloid fate (Rossi et al., 2005). Studies of the cell-extrinsic bone marrow niche indicated that dysfunction of aged marrow macrophages directed HSC platelet bias and that aged mice exhibited markedly more senescent neutrophils than young mice (Frisch et al., 2019).

Most of the traditional knowledge about HSC aging relies on bulk RNA sequencing, which generates an averaged landscape but underestimates the true heterogeneity of cells (Goldman et al., 2019). In addition, the required number of cells for bulk RNA sequencing is large, and it is difficult to generate transcriptome profiles for rare cells including HSCs. With the rapid development of single-cell RNA sequencing technology, the dissection of gene expression has provided unprecedented insights into cellular heterogeneity (Ye et al., 2017). On the one hand, several studies have characterized distinct transcriptome profiles of young and aged HSCs at single-cell resolution and revealed cell-intrinsic differences during HSC aging (Grover et al., 2016; Mann et al., 2018; Frisch et al., 2019). On the other hand, cell-extrinsic mechanisms were also revealed by the single cell transcriptome profiles of young and aged bone marrow niches in different studies (Schaum et al., 2018; Almanzar et al., 2020). However, these results were all based on a single study respectively and lacked reproducibility, consistency and comparability. To overcome these shortcomings, integrated bioinformatics methods should be utilized to further elucidate the mechanisms underlying HSC aging.

In the present study, three independent single-cell transcriptome datasets (Grover et al., 2016; Mann et al., 2018; Frisch et al., 2019) of purified young and aged HSCs were integrated together and cellular heterogeneity analysis was performed to illustrate the differences of subpopulations within HSCs. Besides, common differentially expressed genes (DEGs) between young and aged HSCs were identified and further validated by real-time quantitative reverse transcription PCR (qRT-PCR). Then, several bioinformatics methods, including functional enrichment analysis and protein–protein interaction (PPI) network analysis, were used to profoundly explore the effects and functions of these DEGs. Furthermore, two single-cell transcriptome datasets of the bone marrow niche (Schaum et al., 2018; Almanzar et al., 2020) were also employed to analyze cellular composition and the interactions between HSCs and other cell types in the bone marrow. Finally, to confirm the increased level of inflammation, a Cytoplex Assay was performed to measure the levels of inflammatory cytokines and chemokines in young and aged bone marrow and colony formation unit (CFU) assays were performed to assess the effect of inflammatory cytokines on cellular senescence. Our study elucidated the biological characteristics of HSC aging, and the genes and pathways we identified could be potential biomarkers and targets for the identification and rejuvenation of aged HSCs.

## Materials and Methods

### Dataset Integration, Data Scaling, Dimension Reduction and Clustering

Three datasets of HSC expression profiles from young and aged mice (GSE100906, GSE70657 and GSE100426, dataset information is shown in Supplementary Table S1) were downloaded from the GEO database. To minimize batch effect between datasets, we integrated three datasets following the instructions of Seurat (Butler et al., 2018; Stuart et al., 2019). Briefly, the FindVariableFeatures function was used to identify the top highly variable 2000 genes and the IntegrateData function was used to move the batch effect and correct the datasets (dims = 1:30). Then the ScaleData function of Seurat was used to calculate the scaling expression values and the RunPCA function (npcs = 30) was used to perform a PCA dimensionality reduction by Uniform Manifold Approximation and Projection (UMAP). The FindNeighbors and FindClusters Seurat functions were used to cluster the cells (resolution = 0.8) (See Supplementary Method for details).

### DEGs Identification and Validation by Multi-Cell One-Step PCR Assay

DEG identification was performed using the package DESeq2 (Love et al., 2014). |Foldchange| > 1.5 and *p* < 0.05 were set as the cutoffs to screen for DEGs. Firstly, bone marrow cells were isolated from young (4∼6 weeks, *n* = 3, C57BL/6) and aged (18∼20 months, *n* = 3, C57BL/6) mouse femora and tibiae by flushing bones with 1–3 ml phosphate-buffered saline (PBS) (without Mg^2+^ and Ca^2+^) supplemented with 2% fetal bovine serum. Then the bone marrow cells were resuspended in cold ammonium chloride solution and incubated on ice for 10 min to lyse red blood cells (RBCs). Following RBC lysis, the cells were washed in PBS and then incubated with flow cytometry antibodies (Lineage-AF700, c-kit-PECY5, Sca-1-FITC, CD150-PE, CD48-PECY7) for 30 min. LT-HSCs (Lineage- Sca1+ c-Kit + CD150 + CD48-) were collected with an SH800S Cell Sorter. As the number of sorted LT-HSCs was small, we amplified the transcriptome in single cells by Single Cell Sequence Specific Amplification Kit (P621-01/02, Vazyme, Nanjing, China) and then detected the gene expression by a fluorescent quantitative PCR according to the manufacturer’s instructions. Firstly, the amplification primers of DEGs were mixed together to prepare an Assay Pool (primer concentration 0.1 µM). Then single LT-HSC were transferred into PCR strips with Assay Pool, RT/Taq enzyme and Reaction Mix. After 5 min of freezing at −80°C, the PCR strips were kept at 50°C for about 60 min to perform reverse transcription. Then the cDNA was denatured at 95°C for 15 s, annealed at 60°C for 15 min, and 15 sequence-specific amplification cycles were performed. After pre-amplification of transcriptome, qRT-PCR was performed following the one-step protocol of the SYBR Green master mix (Vazyme) in a Real-time system according to the manufacturer’s instructions (95 C*15 s, 60 C*30 s, 40 cycles). The primer sets for the detection of DEGs are listed in Supplementary Table S3.

### Functional Enrichment and PPI Network Analysis of DEGs

Functional enrichment analysis of DEGs was performed with DAVID website (Huang et al., 2008; Huang et al., 2009). Gene Ontology (GO) term categories included biological process (BP), molecular function (MF) and cellular component (CC). STRING website was used to predict the interactions among DEGs (Szklarczyk et al., 2019). Cytoscape software was applied to establish the PPI network (Shannon et al., 2003) and MCODE was applied to identify the hub genes (Bader and Hogue, 2003). CytoHubba, a plug in Cytoscape, was applied to explore important nodes/hubs in the interactome network (Chin et al., 2014).

### Clustering-Based Analysis of Cell Cycle State

The cell cycle phase (G1, S, or G2M) of each cell was identified by calculating the cell cycle phase score based on canonical markers using the Seurat package (version 3.1) (Butler et al., 2018; Stuart et al., 2019). The canonical cell cycle gene set was defined with “cell cycle process” from the GO annotation and identified as cycling in HeLa cells (Whitfield et al., 2002) and it can be downloaded from GSEA MSigDB version 7.1 (https://www.gsea-msigdb.org/gsea/msigdb) (Subramanian et al., 2005; Liberzon et al., 2015).

### Analysis of Interactions Between HSCs and Surrounding Cells in the Bone Marrow Niche

The single cell transcriptome profiles of young and aged mouse bone marrow based on the microfluidic droplet platform (10x Genomics) were shared by the Tabula Muris Consortium (tabula-muris-senis.ds.czbiohub.org) (Schaum et al., 2018; Almanzar et al., 2020) and can be downloaded from the Gene Expression Omnibus database (GSE109774 and GSE132042). CellphoneDB software was used for cellular interaction analysis (Vento-Tormo et al., 2018; Efremova et al., 2020). Significant ligand–receptor pairs were filtered with *p* < 0.05. To identify which cell population accounted for the difference in inflammatory cytokine levels, CellChat software was applied to analyze outgoing communication patterns of secreting cells (Jin et al., 2021). IdentifyOverExpressedGenes (thresh.*p* = 0.05) and identifyOverExpressedInteractions functions were used to identify over-expressed ligands or receptors and over-expressed ligand-receptor interactions. ComputeCommunProb function (methods for computing the average gene expression per cell group was set as “triMean”) was used to infer the biologically significant cell-cell communication (See Supplementary Method for details).

### Quantitation of Inflammatory Cytokines and Chemokines via a Cytoplex Assay

LEGENDPlex assays (BioLegend) were performed to detect the inflammatory cytokine and chemokine levels of bone marrow from young (4∼6 weeks, *n* = 3, C57BL/6) and aged (18∼20 months, *n* = 3, C57BL/6) mice. The femurs and tibias were cut at both ends, and the bone marrow was flushed out with 1 ml PBS. After 5 min of centrifugation at 400 g, the supernatant was collected. Samples were detected according to the manufacturer’s instructions by flow cytometry.

### Colony Formation Unit Assay

Colony formation unit assays were performed as follows. One hundred FACS-isolated HSCs (Lineage-Sca1+c-Kit+) from young (4∼6 weeks, *n* = 3, C57BL/6) and aged (18∼20 months, *n* = 3, C57BL/6) mice were plated in methylcellulose (M3434 for BFU-E, CFU-GM, CFU-GEMM measurement and M3630 for CFU-B measurement, Stem Cell Technologies) and enumerated on day 10. To test the effect of inflammatory cytokines on hematopoietic cell lineage, either DMSO vehicle or IL-1β (25 ng/ml), TNF-α (1 μg/ml), AS101 (2 μg/ml, IL-1β inhibitor) and R7050 (2 μg/ml, TNF-α receptor antagonist) were added to the methylcellulose media.

## Results

### Cellular Heterogeneity Within Young and Aged HSCs

**FIGURE 1:**
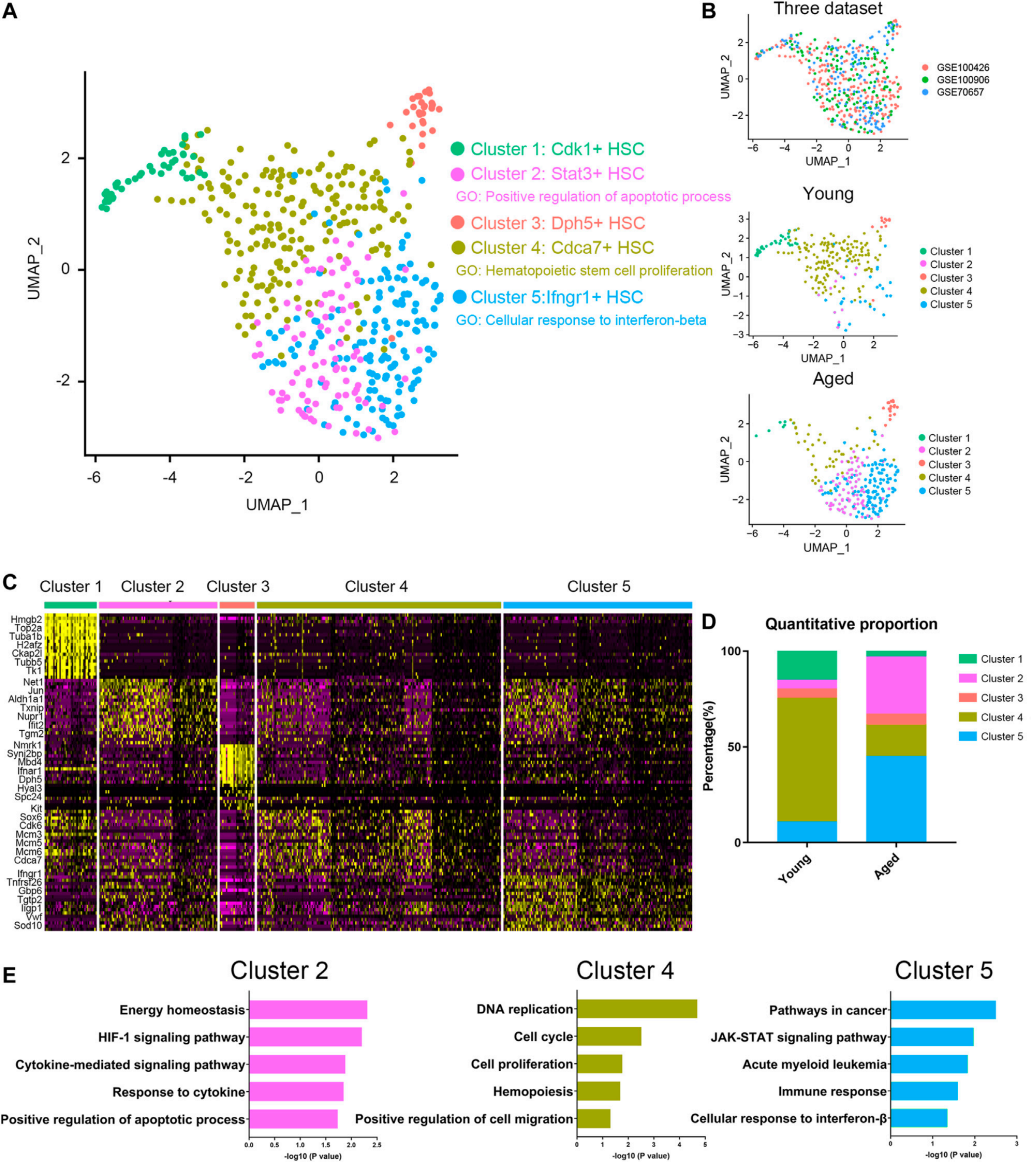
Cellular heterogeneity within young and aged HSCs. **(A,B)** UMAP plot of HSCs analyzed. Five different clusters and cells from three datasets were labeled by color. **(C)** Heatmap of the most significant DEGs in the different clusters. **(D)** Quantitative proportions of different clusters in young and aged HSCs. **(E)** Functional enrichment (biological process and KEGG pathways enrichment) of DEGs of Cluster 2, Cluster 4 and Cluster 5.

To characterize the differences in HSC subpopulations, three datasets (Supplementary Table S1) of HSC single cell expression profiles from young and aged mice were integrated together using Seurat (Butler et al., 2018; Stuart et al., 2019) to correct batch effect. A total of 5 clusters were identified (Figures 1A,B) by using UMAP based on their markers and gene set enrichment analysis (Figures 1C,E). To assess the impact of aging on HSC population, we compared the proportion of different clusters in young and aged HSCs (Figure 1D). Interestingly, Cluster 4, a proliferation-associated cluster marked by Cdca7, decreased significantly upon aging. Besides, Cluster 2 (an apoptosis-associated cluster marked by Stat3) and Cluster 5 (an interferon-associated cluster marked by Ifngr1) were increased upon aging. Ifngr1 encodes the ligand-binding chain of the gamma interferon receptor and IFN-γ has been reported to selectively promote differentiation of myeloid-biased HSCs (Matatall et al., 2014). As a whole, these results highlighted an exhaustion of HSCs being able to give rise to HSC itself without differentiation, which is a hallmark of HSC aging and indicated that Stat3 and Ifngr1 were two markers for accumulation of inflammatory and apoptosis-biased state HSCs.

### Identification of DEGs Between Young and Aged HSCs

**FIGURE 2:**
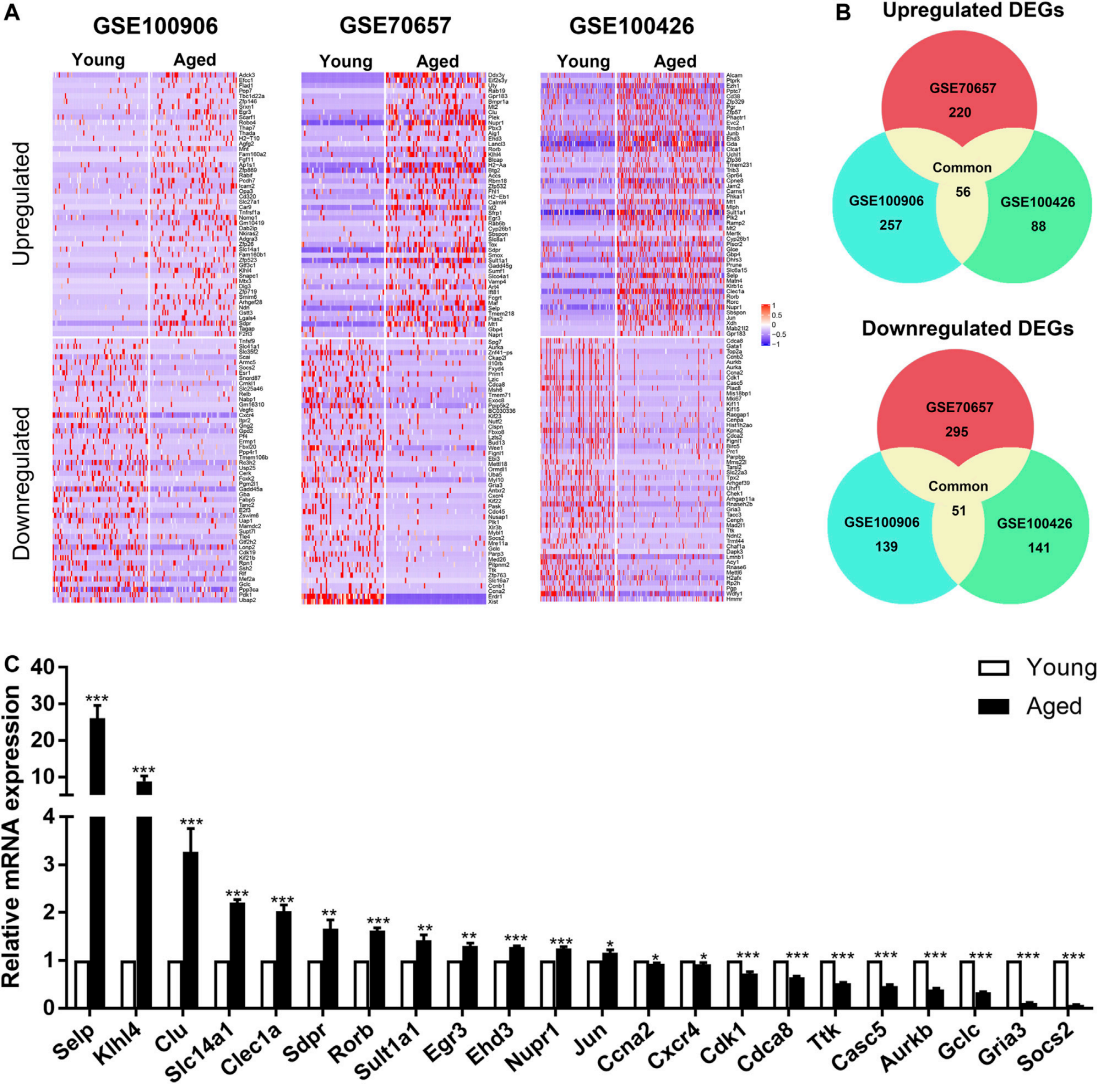
Identification of common differentially expressed genes (DEGs) in 3 independent single-cell transcriptome datasets (GSE100906, GSE70657, and GSE100426). **(A)** Heatmap of the top 100 DEGs (50 upregulated and 50 downregulated genes). **(B)** Common DEGs in the three datasets (56 upregulated and 51 downregulated genes). **(C)** qRT-PCR results of the relative gene expression of top 30 DEGs. Data are presented as the mean ± SEM. *p < 0.05; **p < 0.01; ***p < 0.001.

In order to elucidate the overall alterations of HSC aging, we next analyzed HSCs as a whole. According to the cut-off criterion (|Foldchange| > 1.5 and *p* < 0.05), 453, 614 and 297 DEGs between young and aged HSCs were identified from GSE100906, GSE70657 and GSE100426, respectively (Figure 2A). Common DEGs were defined as genes that were significantly upregulated or downregulated in at least two datasets. By employing integrated bioinformatics analysis, 56 common upregulated genes and 51 common downregulated genes were identified (Figure 2B, Supplementary Table S2). For instance, Birc5 and Kpna2 were downregulated, while Clu, Selp and Sdpr were upregulated in HSCs during aging. To confirm the results of common DEGs, the relative expression levels of top 30 DEGs were analyzed by qRT-PCR (Figure 2C). We found that the PCR results for approximately 75% of the genes were consistent with our bioinformatics analysis (*p* < 0.05).

### Functional Enrichment Analysis of DEGs

**FIGURE 3:**
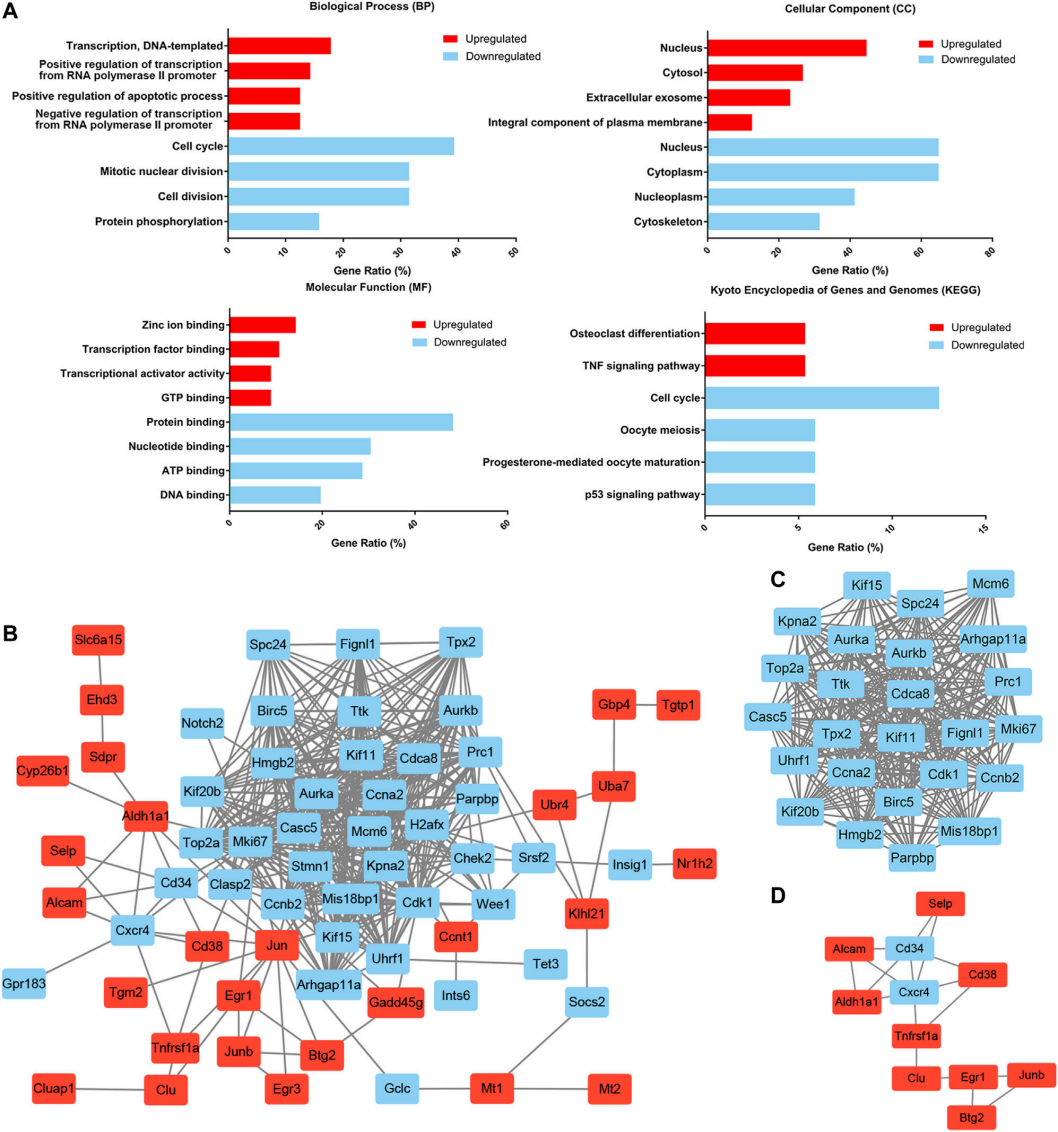
Functional enrichment and protein–protein interaction (PPI) network analysis of common DEGs. **(A)** Functional enrichment of common DEGs. The gene ratio was acquired from the DAVID functional annotation tool. **(B)** The common DEG protein–protein interaction (PPI) network contained 67 nodes and 413 edges. **(C)** Module 1 contained 25 nodes and 286 edges and was mainly involved in the cell cycle. **(D)** Module 2 contained 11 nodes and 17 edges and was mainly involved in cell adhesion molecules and the hematopoietic cell lineage. Upregulated nodes are marked in red and downregulated nodes are marked in blue.

To further delineate the functional changes that occur during HSC aging, functional enrichment of the common DEGs was performed by using the DAVID gene annotation tool (Figure 3A). For BP, the upregulated genes were mainly enriched in transcription, DNA-templated and regulation of transcription from RNA polymerase II promoter, and the downregulated genes were predominantly enriched in cell cycle, mitotic nuclear division, cell division and protein phosphorylation. This was consistent with the previous findings that cell cycle-related genes dominated the transcriptomic variability of aging and that aged HSCs underwent fewer cell divisions than young HSCs (Janzen et al., 2006; Nygren and Bryder, 2008; Kowalczyk et al., 2015). KEGG pathway enrichment analyses revealed that the upregulated DEGs were mainly involved in osteoclast differentiation and TNF signaling pathway, and the downregulated DEGs were mainly involved in cell cycle, oocyte meiosis and p53 signaling pathway. p53 is implicated in regulating HSC aging and quiescence (Dumble et al., 2007) and regulating p53 can help to maintain hematopoietic cells during oxidative stress (Jung et al., 2013). These results highlighted the loss of self-renewal ability and quiescence in aged HSCs.

### PPI Network Analysis of DEGs

To better understand the interactions among the common DEGs, a PPI network with 67 nodes (27 upregulated and 40 downregulated) and 413 edges was generated with the STRING tool (Figure 3B). Two significant modules were also identified based on the degree of importance in Cytotype MCODE. Module 1 contained 25 nodes and 286 edges (Figure 3C), and the expression of all the nodes was downregulated in aged HSCs. KEGG pathway enrichment analyses revealed that the DEGs in Module 1 were mainly enriched in the cell cycle. Module 2 contained 11 nodes and 17 edges (Figure 3D), and the DEGs in Module 2 were mainly enriched in cell adhesion molecules and the hematopoietic cell lineage. In addition, highly connected nodes in the network are likely to have significant functional importance and were defined as hub genes. By utilizing CytoHubba in Cytotype, we identified the top five upregulated hub genes (Jun, Aldh1a1, Egr1, Cd38 and Junb) and the top five downregulated hub genes (Aurka, Ccna2, Ccnb2, Cdk1 and Birc5). The alterations and potential functions of the hub genes were summarized in Table 1.

**TABLE 1:** Alterations and functions of hub genes during HSC the aging process.

Gene	Aliase	Alterations with age	Functions
Aurka	Aurora kinase A	Down	Contributed to the regulation of cell cycle progression
Ccna2	Cyclin-A2	Down	Controlled both the G1/S and the G2/M transition phases of the cell cycle
Ccnb2	G2/mitotic-specific cyclin-B2	Down	Essential for the control of the cell cycle at the G2/M transition
Cdk1	Cyclin-dependent kinase 1	Down	Essential for the control of the eukaryotic cell cycle, promoted G2-M transition, and regulated G1 progress and G1-S transition
Birc5	Baculoviral IAP repeat-containing protein 5	Down	Had dual roles in promoting cell proliferation and preventing apoptosis
Jun	Transcription factor AP-1	Up	Regulated functional development of hematopoietic precursor cells into mature blood cells
Aldh1a1	Retinal dehydrogenase 1	Up	Regulated retinoic acid biosynthesis, the clearance of toxic byproducts of reactive oxygen species and HSC differentiation
Egr1	Early growth response protein 1	Up	Played a role in the regulation of cell survival, proliferation and cell death and in regulating the response to growth factors and DNA damage
Cd38	Cluster of Differentiation 38	Up	Cell adhesion and signal transduction
Junb	Transcription factor jun-B	Up	Involved in regulating gene activity following the primary growth factor response

### Cell Cycle Analysis of HSCs

**FIGURE 4:**
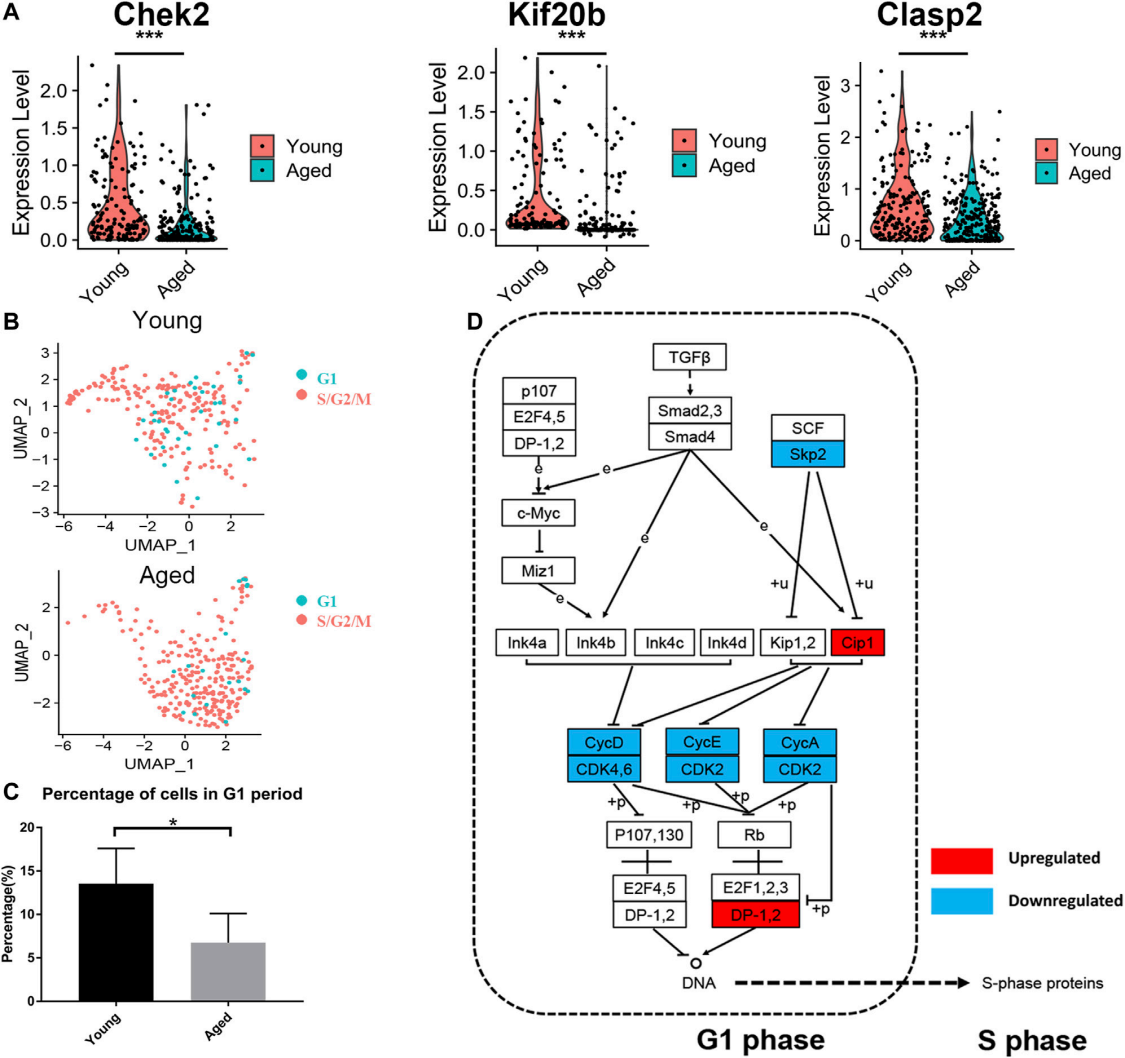
Cell cycle analysis of HSCs. **(A)** Violin plot showing the expression levels of cell cycle-associated genes (Chek2, Kif20b and Clasp2) in young and aged HSCs. **(B)** UMAP plot for young and aged HSCs. **(C)** Histogram of cell frequency in the G1 cluster. Data are presented as the mean ± SEM. *p < 0.05. **(D)** Cell cycle pathway diagram showing alterations in signaling pathway activity. Downregulation of the Skp2-induced signaling pathway (Skp2→Cip1→CycA/CDK2→DP-1) promoted the transition from G1 phase to S phase. Upregulated genes are marked in red and downregulated genes are marked in blue.

As the enrichment analysis of common DEGs and PPI network analysis suggested that the cell cycle was strongly associated with functional decline in aged HSCs, we next compared the expression levels of cell cycle-associated genes. Among 107 DEGs, 24 genes were associated with the cell cycle and most of them (22 genes), including Chek2, Kif20b and Clasp2, were downregulated in at least 2 datasets (Figure 4A). Subsequently, the cell cycle phase of young and aged HSCs was identified by calculating the cell cycle phase score based on canonical markers in both young and aged mice (Figure 4B and Supplementary Figure S1A). The frequency of cells in the G1 cluster significantly decreased in aged versus young cells (13.5 vs. 6.8%; *p* < 0.05, shown in Figure 4C).

To further identify the signaling pathways that drove HSCs to transit through G1 phase quickly, we evaluated genetic alterations in a cell cycle pathway diagram that was publicly available at https://www.genome.jp/kegg (Kanehisa and Goto, 2000). We found that the Skp2-induced signaling pathway (Skp2→Cip1→CycA/CDK2→DP-1) was significantly downregulated during the aging process; this change promoted the synthesis of mRNAs and proteins that are required for DNA synthesis during S phase (Figure 4D).

### Analysis of the Alterations in Bone Marrow During the Aging Process

**FIGURE 5:**
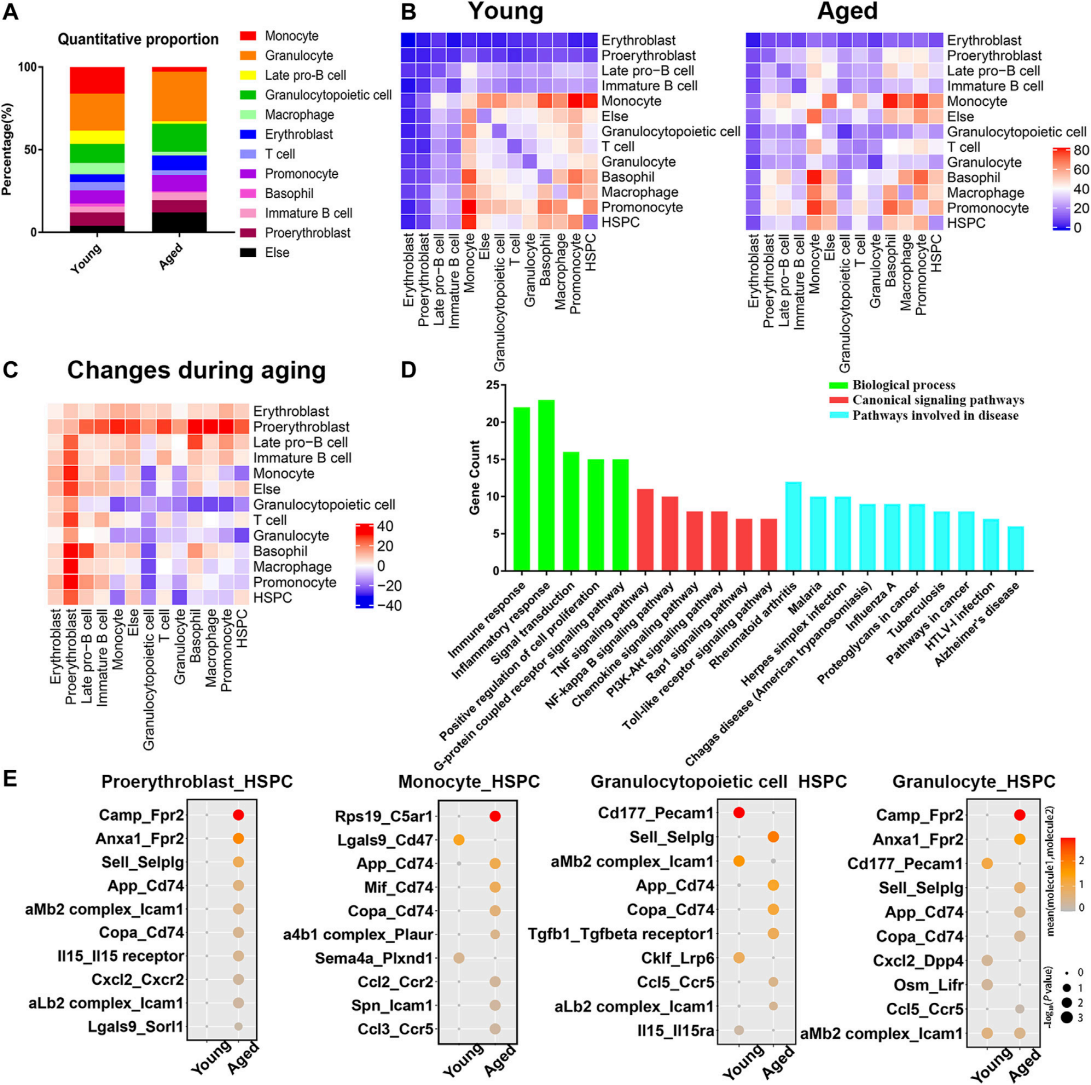
Cellular composition and interactions between HSCs and other cell types in the bone marrow niche. **(A)** Quantitative proportions of different cell types in the bone marrow niche. **(B)** Heatmap showing the number of interactions between different cell types in young and aged bone marrow separately. **(C)** Heatmap showing the differences in the number of interactions (interaction number of aged cells minus the interaction number of young cells). **(D)** Functional enrichment results for differentially expressed ligand–receptor pairs. **(E)** Dot plots showing the ligand–receptor pairs between the ligand of proerythroblasts/monocytes/granulocytopoietic cells/granulocytes and the receptor of HSPCs in young and aged mice. The color of the dots represents the mean expression of each ligand and receptor, and the size of the dots represents −log10 (*p* value).

The PPI network analysis confirmed significant changes of adhesion molecules, highlighting an important role of bone marrow microenvironment in the aging process. Therefore, we reanalyzed two single cell transcriptome profiles of young and aged mouse bone marrow (GSE109774 and GSE132042) and investigated how the cellular composition of the bone marrow changed with age. The percentages of erythroblasts, granulocytopoietic cells and granulocytes increased with age, while the percentages of macrophages, late pro-B cells, monocytes and T cells decreased with age (Figure 5A). Cell-cell communication mediated by receptor-ligand complexes is important for stem cell biological processes (Efremova et al., 2020). To assess alterations in intercellular communication during aging, cellphoneDB software was used to predict interactions among different cell types from the single-cell RNA-seq data (Vento-Tormo et al., 2018; Efremova et al., 2020). Significant predicted interactions were assessed separately for young and aged mice (Figure 5B), and the differences were used to infer alterations in intercellular interactions (Figure 5C). The predicted interactions with hematopoietic stem/progenitor cells (HSPCs) were highest in monocytes and lowest in erythroblasts in both young and aged mice. The comparison between the young and aged groups indicated a significant increase in intercellular communication between HSPCs and proerythroblasts, and a decrease in intercellular communication between HSPCs and monocytes, granulocytopoietic cells and granulocytes (Figure 5C).

As the surrounding cells of HSPCs are potential niche components, the ligands expressed by these populations and the receptors expressed by HSPCs were considered in the downstream analysis. Among a total of 68 heterologous ligand–receptor pairs detected between HSPCs and other cell types, immune response, inflammation response and signal transduction were the predominant biological processes involved (Figure 5D). In addition, ligand–receptor pairs were involved in some canonical signaling pathways (such as the TNF signaling pathway, NF-kappa B signaling pathway and PI3K-Akt signaling pathway) and certain aging-associated diseases (such as Alzheimer’s disease).

The ligand–receptor gene pairs exhibited differential expression patterns between young and aged populations when coupled with HSPCs. Interactions with HSPCs changed significantly in proerythroblasts, monocytes, granulocytopoietic cells and granulocytes, and the significant differentially expressed ligand–receptor gene pairs of these four groups of cells are shown in Figure 5E. Adhesion complexes, such as aMb2 complex-Icam1 and aLb2 complex-Icam1, were differentially expressed during HSC aging. Moreover, inflammatory ligand-receptor pairs, such as CXCL2-CXCR2, IL15-IL15 receptor, CCL2-CCR2 and CCL5-CCR5, were upregulated during HSC aging.

### Analysis of Inflammation Levels in the Bone Marrow Niche

**FIGURE 6:**
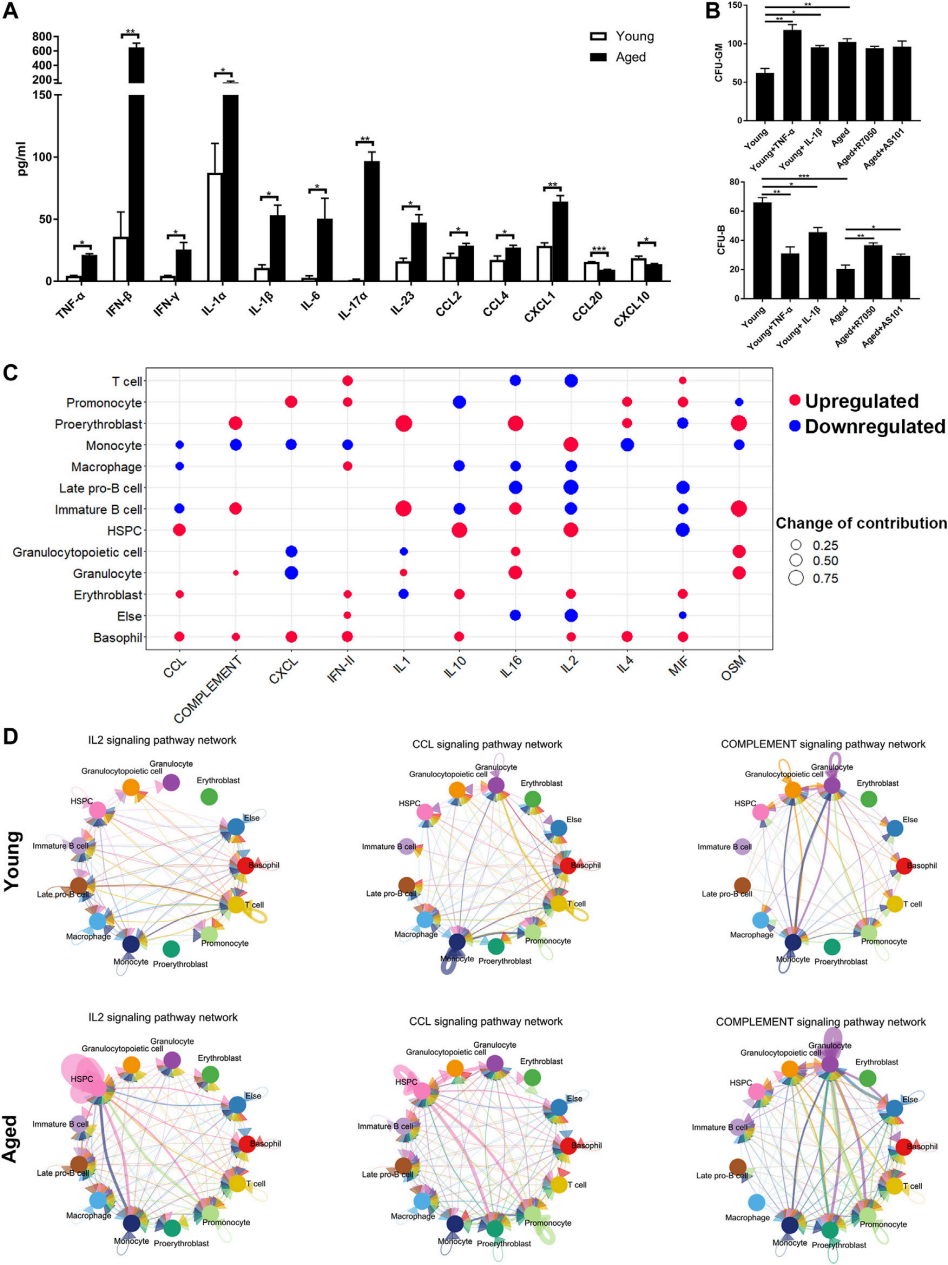
Inflammatory cytokines and chemokines in the bone marrow niche. **(A)** Quantitation of inflammatory cytokines and chemokines in young and aged bone marrow niches via a Cytoplex Assay. Data are presented as the mean ± SEM.*p < 0.1; **p < 0.01; ***p < 0.001. **(B)** Ganulocyte/macrophage (GM) and B cell colony formation was measured for young or aged HSPCs incubated for 10 days in methylcellulose in the presence of either DMSO vehicle or IL-1β (25 ng/ml), TNF-α (1 μg/ml), AS101 (2 μg/ml, IL-1β inhibitor) and R7050 (2 μg/ml, TNF-α receptor antagonist). Graph shows mean ± SEM. **(C)** Dot plots showing the differences in the contribution to different inflammatory signaling pathways. Upregulated cytokines and chemokines are marked in red and downregulated cytokines and chemokines are marked in blue. The size of the dots represents the change in contribution. **(D)** Circle plot of the cell-cell communication network showing that the inflammatory pathway network (IL2, CCL and complement signaling pathways) became more complex and interconnected in the aged bone marrow niche.

The upregulated inflammatory ligand-receptor pairs in aged HSCs suggested increasing inflammation levels in the bone marrow niche during aging. To test our hypothesis, a Cytoplex Assay was performed to measure the levels of inflammatory cytokines and chemokines in the bone marrow. Most inflammatory cytokines and chemokines, including TNF-α, IFN-β, IFN-γ, IL-1α, IL-1β, IL-6, IL-17α, IL-23, CCL2, CCL4 and CXCL1, were upregulated in aged bone marrow niche, while CCL20 and CXCL10 were downregulated in the aged bone marrow niche (Figure 6A). To assess the effect of inflammatory cytokines on cellular senescence, HSCs were cultured in methylcellulose with either inflammatory cytokines or their inhibitors. TNF-α and IL-1β promoted myeloid-biased differentiation and inflammatory pathway blockade may rejuvenate aged HSC functions and increase B cell output (Figure 6B).

To further determine which cell population accounted for the difference in inflammatory cytokine levels, the outgoing communication patterns of secreting cells were analyzed (Figure 6C). The secretion of HSPCs, erythroblasts and basophils contributed to high CCL levels in the aged bone marrow niche, while the secretion of T cells, promonocytes, macrophages, erythroblasts, and basophils contributed to high IFN-γ levels. In addition, the secretion of some interleukins and complement factors was upregulated in proerythroblasts and immature B cells. Interestingly, the levels of inflammatory cytokines secreted by HSPCs, including CCL and IL2, were increased, highlighting the important role of autocrine signaling during aging process. Circle plots of the cell-cell communication network also revealed that inflammatory pathway networks (such as IL2, CCL and complement; Figure 6D, Supplementary Figure S1B) became more interconnected during aging, with some ligand-receptor communication enhanced significantly (such as IL2-IL2RB/IL2RG and CCL5-CCR1; Supplementary Figure S1C).

## Discussion

HSC aging is a comprehensive result of both intrinsic and extrinsic factors. To illustrate the biological characteristics of HSC aging, we integrated 3 independent single-cell transcriptome datasets of HSCs together and identified cellular heterogeneity within HSCs. Common DEGs between young and aged HSCs were identified and confirmed by qRT-PCR, and several bioinformatics methods were employed to profoundly explore the biological functions of DEGs. Besides, we compared the cellular composition of the bone marrow, analyzed intercellular communication between HSCs and other cell types and identified important differentially expressed ligand-receptor pairs. Furthermore, we measured the levels of some inflammatory cytokines in the bone marrow niche and determined which cell population accounted for high inflammatory cytokine levels in aged bone marrow.

First, we identified two HSCs subsets (Stat3+ apoptosis-associated HSCs and Ifngr1+ interferon-associated HSCs) that accumulated significantly with aging. Consistently, it was reported that Stat3 was upregulated in aged HSCs and that activation of JAK/STAT pathway led to a functionally impairment of aged HSCs (Kirschner et al., 2017). Besides, functional enrichment of DEGs revealed that the downregulated genes were predominantly involved in the cell cycle (Figure 3). Consistent with our findings, previous studies have shown that the cell cycle activity of HSCs declined during the aging process, with aged HSCs undergoing fewer cell divisions than young HSCs (Janzen et al., 2006; Nygren and Bryder, 2008). Further analysis revealed that the number of cells in G1 phase decreased in aged HSCs (Figure 4) and that downregulation of the Skp2-induced signaling pathway (Skp2→Cip1→CycA/CDK2→DP-1) in aged HSCs promoted the transition from G1 phase to S phase. Skp2 was reported to specifically interact with p27Kip1 to promote its ubiquitination and play a vital role in the cell cycle progression from G1 to S phase (Imaki et al., 2003; Wei et al., 2004). Recently, a study on endothelial progenitor cells reported that depletion of Skp2 generated a senescent bioenergetic profile, whereas adenoviral-mediated Skp2 expression reversed this senescence (Wang et al., 2020). Therefore, the Skp2-induced signaling pathway may promote alterations in the distribution of cells in different cell cycle stages and activation of this signaling pathway may promote rejuvenation of aged HSCs.

In addition to the cell cycle, the DEGs were predominantly involved in the TNF signaling pathway (Figure 3D). Consistent with this, some inflammatory ligand-receptor pairs (such as CXCL2-CXCR2, CCL2-CCR2 and CCL5-CCR5) and most of the inflammatory cytokines and chemokines in the bone marrow niche were upregulated during aging (Figure 6A). CCL6, which was responsible for the elevated reactive oxygen species in HSCs, could impair HSC homeostasis and that blockage of CCL6 restored the reconstitution ability of HSCs (Zhang et al., 2018). These results emphasized the importance of chemokines on HSC aging and regulation of these chemokines has therapeutic application value. Interestingly, the levels of inflammatory signaling molecules secreted by HSPCs were increased, highlighting the important role of autocrine signaling during the aging process. Consistently, it was reported that direct Toll-like receptor activation in HSPCs resulted in the production of proinflammatory cytokines such as IL-6, in either a paracrine or an autocrine manner, and enhanced proliferation and myeloid differentiation under neutropenic conditions *in vivo* (Zhao et al., 2014). Taken together, these findings indicated that both autocrine signals from HSPCs themselves and paracrine signals played important roles in the high levels of inflammation in the aged bone marrow niche.

Compared with the previous studies based on a single dataset, our integrated bioinformatics analysis took advantage of multiple transcriptome datasets to illustrate the commonness of HSC aging, improving reproducibility and reliability. On the one hand, most of the DEGs and the signaling pathway we identified were consistent with the previous experiment-based studies. More importantly, we yielded some new insights about the mechanisms of HSC aging, including potential pathways driving alterations about cell cycle. Besides, although previous reports have demonstrated that a state of chronic inflammation is correlated with aging and is a strong risk factor for the occurrence of many chronic diseases (Franceschi et al., 2007; Franceschi and Campisi, 2014; Shavlakadze et al., 2019; He et al., 2020), the exact mechanisms underlying the increase in inflammatory factors are not completely understood. By utilizing single cell transcriptome data, expression of inflammatory cytokines in different cell populations can be revealed. For instance, the secretion of HSPCs, erythroblasts and basophils contributed to high CCL levels in the aged bone marrow niche, while the secretion of T cells, promonocytes, macrophages, erythroblasts, and basophils contributed to high IFN-γ levels. Collectively, our study elucidated the biological characteristics of HSC aging. Moreover, the genes and pathways we identified could be potential biomarkers and targets for the identification and rejuvenation of aged HSCs.

## Data Availability

Publicly available datasets were analyzed in this study. This data can be found here: https://www.ncbi.nlm.nih.gov/geo/ (GSE100906, GSE70657, GSE100426, GSE109774 and GSE132042).

## Abbreviations

HSC, hematopoietic stem cell; DEG, differentially expressed gene; TNF, tumor necrosis factor; qRT-PCR, real-time quantitative reverse transcription PCR; PPI, protein–protein interaction; CFU, colony formation unit; HSPC, hematopoietic stem/progenitor cell; GEO, gene expression omnibus; GO, gene ontology; KEGG, kyoto encyclopedia of genes and genomes; BP, biological process; MF, molecular function; CC, cellular component.

## References

[B1] Almanzar N. Almanzar N. Antony J. Baghel A. S. Bakerman I. Bansal I. (2020). A Single-Cell Transcriptomic Atlas Characterizes Ageing Tissues in the Mouse. Nature 583, 590–595. 10.1038/s41586-020-2496-1 32669714 PMC8240505

[B2] Bader G. D. Hogue C. W. (2003). An Automated Method for Finding Molecular Complexes in Large Protein Interaction Networks. BMC Bioinformatics 4, 2. 10.1186/1471-2105-4-2 12525261 PMC149346

[B3] Butler A. Hoffman P. Smibert P. Papalexi E. Satija R. (2018). Integrating Single-Cell Transcriptomic Data across Different Conditions, Technologies, and Species. Nat. Biotechnol. 36, 411–420. 10.1038/nbt.4096 29608179 PMC6700744

[B4] Chin C. H. Chen S. H. Wu H. H. Ho C. W. Ko M. T. Lin C. Y. (2014). cytoHubba: Identifying Hub Objects and Sub-networks from Complex Interactome. BMC Syst. Biol. 8 (Suppl. 4), S11. 10.1186/1752-0509-8-S4-S11 25521941 PMC4290687

[B5] Dumble M. Moore L. Chambers S. M. Geiger H. Van Zant G. Goodell M. A. (2007). The Impact of Altered P53 Dosage on Hematopoietic Stem Cell Dynamics during Aging. Blood 109, 1736–1742. 10.1182/blood-2006-03-010413 17032926 PMC1794064

[B6] Dykstra B. Olthof S. Schreuder J. Ritsema M. de Haan G. (2011). Clonal Analysis Reveals Multiple Functional Defects of Aged Murine Hematopoietic Stem Cells. J. Exp. Med. 208, 2691–2703. 10.1084/jem.20111490 22110168 PMC3244040

[B7] Efremova M. Vento-Tormo M. Teichmann S. A. Vento-Tormo R. (2020). CellPhoneDB: Inferring Cell-Cell Communication from Combined Expression of Multi-Subunit Ligand-Receptor Complexes. Nat. Protoc. 15, 1484–1506. 10.1038/s41596-020-0292-x 32103204

[B8] Franceschi C. Campisi J. (2014). Chronic Inflammation (Inflammaging) and its Potential Contribution to Age-Associated Diseases. J. Gerontol. A. Biol. Sci. Med. Sci. 69 (Suppl. 1), S4–S9. 10.1093/gerona/glu057 24833586

[B9] Franceschi C. Capri M. Monti D. Giunta S. Olivieri F. Sevini F. (2007). Inflammaging and Anti-inflammaging: a Systemic Perspective on Aging and Longevity Emerged from Studies in Humans. Mech. Ageing Develop. 128, 92–105. 10.1016/j.mad.2006.11.016 17116321

[B10] Frisch B. J. Hoffman C. M. Latchney S. E. LaMere M. W. Myers J. Ashton J. (2019). Aged Marrow Macrophages Expand Platelet-Biased Hematopoietic Stem Cells via interleukin-1B. JCI insight 4, e124213. 10.1172/jci.insight.124213 PMC6542605 30998506

[B11] Goldman S. L. MacKay M. Afshinnekoo E. Melnick A. M. Wu S. Mason C. E. (2019). The Impact of Heterogeneity on Single-Cell Sequencing. Front. Genet. 10, 8. 10.3389/fgene.2019.00008 30881372 PMC6405636

[B12] Grover A. Sanjuan-Pla A. Thongjuea S. Carrelha J. Giustacchini A. Gambardella A. (2016). Single-cell RNA Sequencing Reveals Molecular and Functional Platelet Bias of Aged Haematopoietic Stem Cells. Nat. Commun. 7, 11075. 10.1038/ncomms11075 27009448 PMC4820843

[B13] He H. Xu P. Zhang X. Liao M. Dong Q. Cong T. (2020). Aging-induced IL27Ra Signaling Impairs Hematopoietic Stem Cells. Blood 136, 183–198. 10.1182/blood.2019003910 32305041

[B14] Huang D. W. Sherman B. T. Lempicki R. A. (2008). Bioinformatics Enrichment Tools: Paths toward the Comprehensive Functional Analysis of Large Gene Lists. Nucleic Acids Res. 37, 1–13. 10.1093/nar/gkn923 19033363 PMC2615629

[B15] Huang D. W. Sherman B. T. Lempicki R. A. (2009). Systematic and Integrative Analysis of Large Gene Lists Using DAVID Bioinformatics Resources. Nat. Protoc. 4, 44–57. 10.1038/nprot.2008.211 19131956

[B16] Imaki H. Nakayama K. Delehouzee S. Handa H. Kitagawa M. Kamura T. (2003). Cell Cycle-dependent Regulation of the Skp2 Promoter by GA-binding Protein. Cancer Res. 63, 4607–4613. 12907639

[B17] Janzen V. Forkert R. Fleming H. E. Saito Y. Waring M. T. Dombkowski D. M. (2006). Stem-cell Ageing Modified by the Cyclin-dependent Kinase Inhibitor p16INK4a. Nature 443, 421–426. 10.1038/nature05159 16957735

[B18] Jin S. Guerrero-Juarez C. F. Zhang L. Chang I. Ramos R. Kuan C.-H. (2021). Inference and Analysis of Cell-Cell Communication Using CellChat. Nat. Commun. 12, 1088. 10.1038/s41467-021-21246-9 33597522 PMC7889871

[B19] Jung H. Kim M. J. Kim D. O. Kim W. S. Yoon S.-J. Park Y.-J. (2013). TXNIP Maintains the Hematopoietic Cell Pool by Switching the Function of P53 under Oxidative Stress. Cel Metab. 18, 75–85. 10.1016/j.cmet.2013.06.002 23823478

[B20] Kanehisa M. Goto S. (2000). KEGG: Kyoto Encyclopedia of Genes and Genomes. Nucleic Acids Res. 28, 27–30. 10.1093/nar/28.1.27 10592173 PMC102409

[B21] Kirschner K. Chandra T. Kiselev V. Flores-Santa Cruz D. Macaulay I. C. Park H. J. (2017). Proliferation Drives Aging-Related Functional Decline in a Subpopulation of the Hematopoietic Stem Cell Compartment. Cel Rep. 19, 1503–1511. 10.1016/j.celrep.2017.04.074 PMC5457484 28538171

[B22] Kowalczyk M. S. Tirosh I. Heckl D. Rao T. N. Dixit A. Haas B. J. (2015). Single-cell RNA-Seq Reveals Changes in Cell Cycle and Differentiation Programs upon Aging of Hematopoietic Stem Cells. Genome Res. 25, 1860–1872. 10.1101/gr.192237.115 26430063 PMC4665007

[B23] Li X. Zeng X. Xu Y. Wang B. Zhao Y. Lai X. (2020). Mechanisms and Rejuvenation Strategies for Aged Hematopoietic Stem Cells. J. Hematol. Oncol. 13, 31. 10.1186/s13045-020-00864-8 32252797 PMC7137344

[B24] Liang Y. Van Zant G. Szilvassy S. J. (2005). Effects of Aging on the Homing and Engraftment of Murine Hematopoietic Stem and Progenitor Cells. Blood 106, 1479–1487. 10.1182/blood-2004-11-4282 15827136 PMC1895199

[B25] Liberzon A. Birger C. Thorvaldsdóttir H. Ghandi M. Mesirov J. P. Tamayo P. (2015). The Molecular Signatures Database Hallmark Gene Set Collection. Cel Syst. 1, 417–425. 10.1016/j.cels.2015.12.004 PMC4707969 26771021

[B26] Love M. I. Huber W. Anders S. (2014). Moderated Estimation of Fold Change and Dispersion for RNA-Seq Data with DESeq2. Genome Biol. 15, 550. 10.1186/s13059-014-0550-8 25516281 PMC4302049

[B27] Mann M. Mehta A. de Boer C. G. Kowalczyk M. S. Lee K. Haldeman P. (2018). Heterogeneous Responses of Hematopoietic Stem Cells to Inflammatory Stimuli Are Altered with Age. Cel Rep. 25, 2992–3005. e2995. 10.1016/j.celrep.2018.11.056 PMC6424521 30540934

[B28] Matatall K. A. Shen C.-C. Challen G. A. King K. Y. (2014). Type II Interferon Promotes Differentiation of Myeloid-Biased Hematopoietic Stem Cells. Stem Cells 32, 3023–3030. 10.1002/stem.1799 25078851 PMC4198460

[B29] Nygren J. M. Bryder D. (2008). A Novel Assay to Trace Proliferation History *In Vivo* Reveals that Enhanced Divisional Kinetics Accompany Loss of Hematopoietic Stem Cell Self-Renewal. PLoS One 3, e3710. 10.1371/journal.pone.0003710 19002266 PMC2580029

[B30] Rossi D. J. Bryder D. Zahn J. M. Ahlenius H. Sonu R. Wagers A. J. (2005). Cell Intrinsic Alterations Underlie Hematopoietic Stem Cell Aging. Proc. Natl. Acad. Sci. 102, 9194–9199. 10.1073/pnas.0503280102 15967997 PMC1153718

[B31] Schaum N. Karkanias J. Neff N. F. May A. P. Quake S. R. Wyss-Coray T. (2018). Single-cell Transcriptomics of 20 Mouse Organs Creates a Tabula Muris. Nature 562, 367–372. 10.1038/s41586-018-0590-4 30283141 PMC6642641

[B32] Shannon P. Markiel A. Ozier O. Baliga N. S. Wang J. T. Ramage D. (2003). Cytoscape: a Software Environment for Integrated Models of Biomolecular Interaction Networks. Genome Res. 13, 2498–2504. 10.1101/gr.1239303 14597658 PMC403769

[B33] Shavlakadze T. Morris M. Fang J. Wang S. X. Zhu J. Zhou W. (2019). Age-Related Gene Expression Signature in Rats Demonstrate Early, Late, and Linear Transcriptional Changes from Multiple Tissues. Cel Rep. 28, 3263–3273. e3263. 10.1016/j.celrep.2019.08.043 31533046

[B34] Stuart T. Butler A. Hoffman P. Hafemeister C. Papalexi E. Mauck W. M. 3rd (2019). Comprehensive Integration of Single-Cell Data. Cell 177, 1888–1902. 10.1016/j.cell.2019.05.031 31178118 PMC6687398

[B35] Subramanian A. Tamayo P. Mootha V. K. Mukherjee S. Ebert B. L. Gillette M. A. (2005). Gene Set Enrichment Analysis: a Knowledge-Based Approach for Interpreting Genome-wide Expression Profiles. Proc. Natl. Acad. Sci. 102, 15545–15550. 10.1073/pnas.0506580102 16199517 PMC1239896

[B36] Szklarczyk D. Gable A. L. Lyon D. Junge A. Wyder S. Huerta-Cepas J. (2019). STRING V11: Protein-Protein Association Networks with Increased Coverage, Supporting Functional Discovery in Genome-wide Experimental Datasets. Nucleic Acids Res. 47, D607–d613. 10.1093/nar/gky1131 30476243 PMC6323986

[B37] Vento-Tormo R. Efremova M. Botting R. A. Turco M. Y. Vento-Tormo M. Meyer K. B. (2018). Single-cell Reconstruction of the Early Maternal-Fetal Interface in Humans. Nature 563, 347–353. 10.1038/s41586-018-0698-6 30429548 PMC7612850

[B38] Wang H.-H. Lee Y.-N. Su C.-H. Shu K.-T. Liu W.-T. Hsieh C.-L. (2020). S-phase Kinase-Associated Protein-2 Rejuvenates Senescent Endothelial Progenitor Cells and Induces Angiogenesis *In Vivo* . Sci. Rep. 10, 6646. 10.1038/s41598-020-63716-y 32313103 PMC7171137

[B39] Wei W. Ayad N. G. Wan Y. Zhang G.-J. Kirschner M. W. Kaelin W. G. Jr. (2004). Degradation of the SCF Component Skp2 in Cell-Cycle Phase G1 by the Anaphase-Promoting Complex. Nature 428, 194–198. 10.1038/nature02381 15014503

[B40] Whitfield M. L. Sherlock G. Saldanha A. J. Murray J. I. Ball C. A. Alexander K. E. (2002). Identification of Genes Periodically Expressed in the Human Cell Cycle and Their Expression in Tumors. MBoC 13, 1977–2000. 10.1091/mbc.02-02-0030 12058064 PMC117619

[B41] Ye F. Huang W. Guo G. (2017). Studying Hematopoiesis Using Single-Cell Technologies. J. Hematol. Oncol. 10, 27. 10.1186/s13045-017-0401-7 28109325 PMC5251333

[B42] Zhang C. Yi W. Li F. Du X. Wang H. Wu P. (2018). Eosinophil-derived CCL-6 Impairs Hematopoietic Stem Cell Homeostasis. Cell Res 28, 323–335. 10.1038/cr.2018.2 29327730 PMC5835778

[B43] Zhao J. L. Ma C. O’Connell R. M. Mehta A. DiLoreto R. Heath J. R. (2014). Conversion of Danger Signals into Cytokine Signals by Hematopoietic Stem and Progenitor Cells for Regulation of Stress-Induced Hematopoiesis. Cell Stem Cell 14, 445–459. 10.1016/j.stem.2014.01.007 24561084 PMC4119790

