# Supplementary material for: Integrated Single-Cell Bioinformatics Analysis Reveals Intrinsic and Extrinsic Biological Characteristics of Hematopoietic Stem Cell Aging
Source: Front Genet. 2021 Oct 19;12:745786. doi: 10.3389/fgene.2021.745786 (PMC8560737; doi:10.3389/fgene.2021.745786)
Supplement: Supplementary file 1 [file Table1.DOCX]

Supplementary Table 1: Information of HSC expression profile datasets.

| Dataset | Markers of HSC | Age of mice; cell number | Method | Reference |
| --- | --- | --- | --- | --- |
| GSE100906 | Lineage-Sca1+c-Kit+ CD150+CD48-Flt3- | Young mice: 1.5-2 months; 78 Aged mice: 20-24 months;71 | Fluidigm C1 Single-cell AutoPrep system | Frisch et al., 2019 |
| GSE70657 | Lineage-Sca1+c-Kit+ CD150+CD48– | Young mice: 2-3 months; 61 Aged mice: 20-25 months; 74 | Fluidigm C1 Single-cell AutoPrep system | Grover et al., 2016 |
| GSE100426 | Lineage-Sca1+c-Kit+ CD150+CD48- | Young mice: 2-3 months; 128 Aged mice: 20-24 months; 192 | SMART-Seq2 | Mann et al., 2018 |
