# Supplementary figures and images for: Integrated Single-Cell Bioinformatics Analysis Reveals Intrinsic and Extrinsic Biological Characteristics of Hematopoietic Stem Cell Aging
Source: Front Genet. 2021 Oct 19;12:745786. doi: 10.3389/fgene.2021.745786 (PMC8560737; doi:10.3389/fgene.2021.745786)

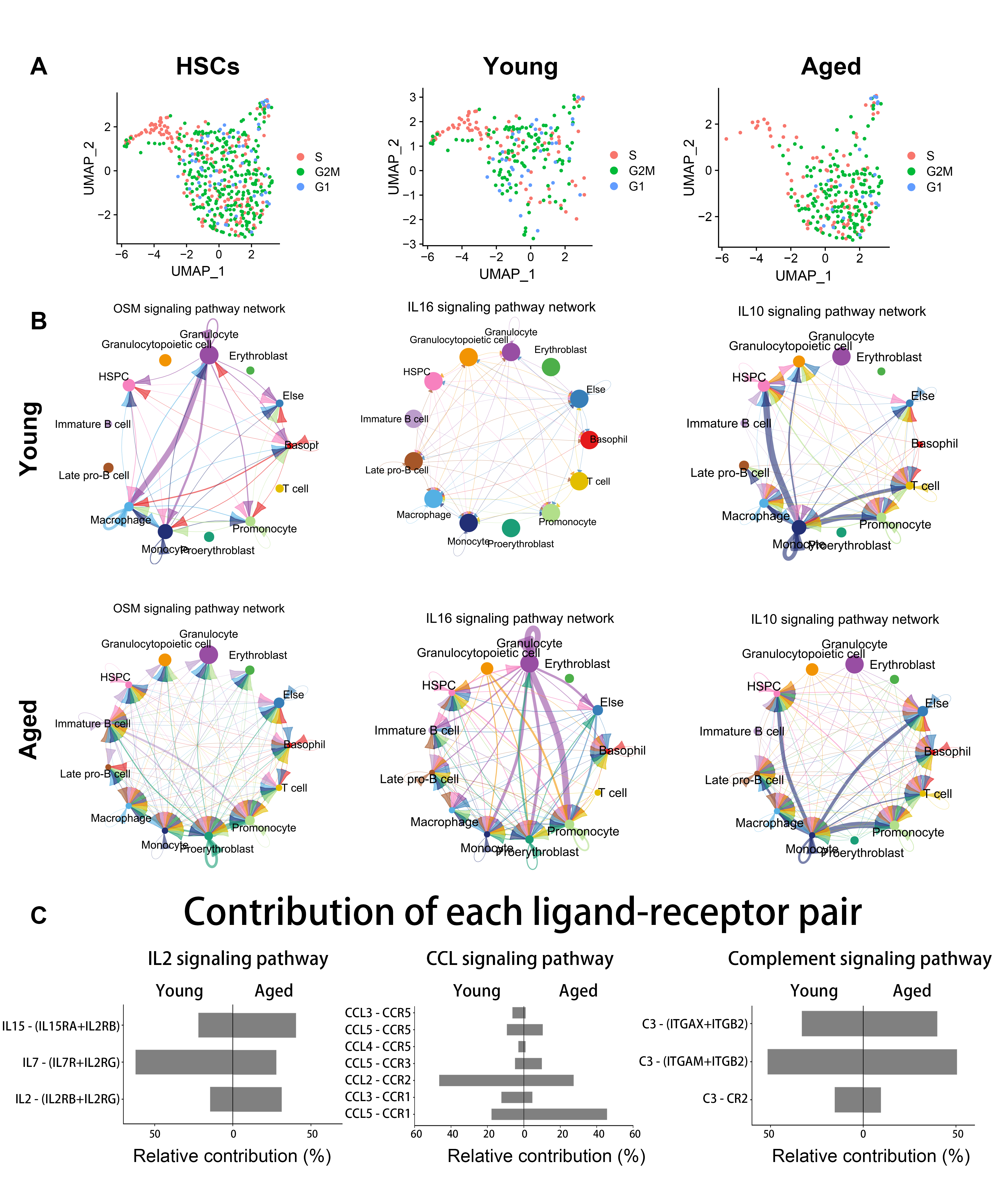

Supplement: Supplementary file 2 [file Image1.TIF]
