# Supplementary material for: Integrated Single-Cell Bioinformatics Analysis Reveals Intrinsic and Extrinsic Biological Characteristics of Hematopoietic Stem Cell Aging
Source: Front Genet. 2021 Oct 19;12:745786. doi: 10.3389/fgene.2021.745786 (PMC8560737; doi:10.3389/fgene.2021.745786)
Supplement: Supplementary file 3 [file Table2.DOCX]

| Supplementary Table 2: Common differentially expressed genes (DEGs) list. | | |
| --- | --- | --- |
| **Gene** | **Aliase** | **Alterations** |
| Adck3 | Atypical kinase COQ8A | Upregulated |
| Alcam | CD166 antigen | Upregulated |
| Aldh1a1 | Retinal dehydrogenase 1 | Upregulated |
| Arf2 | ADP-ribosylation factor 2 | Upregulated |
| Asrgl1 | Isoaspartyl peptidase/L-asparaginase | Upregulated |
| Btg2 | Protein BTG2 | Upregulated |
| Ccnt1 | Cyclin-T1 | Upregulated |
| Cd38 | Cluster of Differentiation 38 | Upregulated |
| Cirbp | Cold-inducible RNA-binding protein | Upregulated |
| Clca1 | Calcium-activated chloride channel regulator 3A-1 | Upregulated |
| Clec1a | C-type lectin domain family 1 | Upregulated |
| Clu | Clusterin | Upregulated |
| Cluap1 | Clusterin-associated protein 1 | Upregulated |
| Cpne8 | Copine-8 | Upregulated |
| Cyp26b1 | Cytochrome P450 26B1 | Upregulated |
| Cysltr2 | Cysteinyl leukotriene receptor 2 | Upregulated |
| Cytip | Cytohesin-interacting protein | Upregulated |
| Egr1 | Early growth response protein 1 | Upregulated |
| Egr3 | Early growth response protein 3 | Upregulated |
| Ehd3 | EH domain-containing protein 3 | Upregulated |
| Eif1a | Eukaryotic translation initiation factor 1A | Upregulated |
| Gadd45g | Growth arrest and DNA damage-inducible protein GADD45 gamma | Upregulated |
| Gbp4 | Guanylate binding protein 4 | Upregulated |
| Gda | Guanine deaminase | Upregulated |
| Gpr183 | G-protein coupled receptor 183 | Upregulated |
| Jam2 | Junctional adhesion molecule B | Upregulated |
| Jun | Transcription factor AP-1 | Upregulated |
| Junb | Transcription factor jun-B | Upregulated |
| Klhl21 | Kelch-like protein 21 | Upregulated |
| Klhl4 | Kelch-like 4 (Drosophila) | Upregulated |
| Med12l | Mediator of RNA polymerase II transcription subunit 12-like protein | Upregulated |
| Mt1 | Metallothionein-1 | Upregulated |
| Mt2 | Metallothionein-2 | Upregulated |
| Muc13 | Mucin-13 | Upregulated |
| Nr1h2 | Oxysterols receptor LXR-beta | Upregulated |
| Nupr1 | Nuclear protein 1 | Upregulated |
| Oxr1 | Oxidation resistance protein 1 | Upregulated |
| Pam | Peptidyl-glycine alpha-amidating monooxygenase | Upregulated |
| Phactr1 | Phosphatase and actin regulator 1 | Upregulated |
| Plscr1 | Phospholipid scramblase 1 | Upregulated |
| Prtn3 | Myeloblastin | Upregulated |
| Rorb | Nuclear receptor ROR-beta | Upregulated |
| Sbspon | Somatomedin-B and thrombospondin type-1 domain-containing protein | Upregulated |
| Sdpr | Caveolae-associated protein 2 | Upregulated |
| Selm | Selenoprotein M | Upregulated |
| Selp | P-selectin | Upregulated |
| Slc14a1 | Urea transporter 1 | Upregulated |
| Slc6a15 | Sodium-dependent neutral amino acid transporter B(0)AT2 | Upregulated |
| Sult1a1 | Sulfotransferase 1A1 | Upregulated |
| Tgm2 | Protein-glutamine gamma-glutamyltransferase 2 | Upregulated |
| Tgtp1 | T-cell-specific guanine nucleotide triphosphate-binding protein 1 | Upregulated |
| Tmem176a | Transmembrane protein 176A | Upregulated |
| Tnfrsf1a | Tumor necrosis factor receptor superfamily member 1A | Upregulated |
| Tsc22d3 | TSC22 domain family protein 3 | Upregulated |
| Uba7 | Ubiquitin-like modifier activating enzyme 7 | Upregulated |
| Ubr4 | E3 ubiquitin-protein ligase UBR4 | Upregulated |
| Arhgap11a | Rho GTPase activating protein 11A | Downregulated |
| Aurka | Aurora kinase A | Downregulated |
| Aurkb | Aurora kinase B | Downregulated |
| Birc5 | Baculoviral IAP repeat-containing protein 5 | Downregulated |
| Casc5 | Kinetochore scaffold 1 | Downregulated |
| Ccna2 | Cyclin-A2 | Downregulated |
| Ccnb2 | G2/mitotic-specific cyclin-B2 | Downregulated |
| Cd34 | Hematopoietic progenitor cell antigen CD34 | Downregulated |
| Cdc42se2 | CDC42 small effector protein 2 | Downregulated |
| Cdca8 | Cell Division Cycle Associated 8 | Downregulated |
| Cdk1 | Cyclin-dependent kinase 1 | Downregulated |
| Chek2 | Serine/threonine-protein kinase Chk2 | Downregulated |
| Clasp2 | CLIP-associating protein 2 | Downregulated |
| Cxcr4 | C-X-C chemokine receptor type 4 | Downregulated |
| Dynll1 | Dynein light chain 1 | Downregulated |
| Fignl1 | Fidgetin-like protein 1 | Downregulated |
| Gclc | Glutamate-cysteine ligase | Downregulated |
| Gria3 | Glutamate receptor 3 | Downregulated |
| H2afx | Histone H2AX | Downregulated |
| Hmgb2 | High mobility group protein B2 | Downregulated |
| Hnrnpul2 | Heterogeneous nuclear ribonucleoprotein U-like 2 | Downregulated |
| Ikzf2 | Zinc finger protein Helios | Downregulated |
| Insig1 | Insulin-induced gene 1 protein | Downregulated |
| Ints6 | Integrator complex subunit 6 | Downregulated |
| Itpripl1 | Inositol 1 | Downregulated |
| Khsrp | Far upstream element-binding protein 2 | Downregulated |
| Kif11 | Kinesin-like protein KIF11 | Downregulated |
| Kif15 | Kinesin-like protein KIF15 | Downregulated |
| Kif20b | Kinesin-like protein KIF20B | Downregulated |
| Kpna2 | Karyopherin (importin) alpha 2 | Downregulated |
| Marcksl1 | MARCKS-related protein | Downregulated |
| Mcm6 | Minichromosome Maintenance Complex Component 6 | Downregulated |
| Mis18bp1 | Mis18-binding protein 1 | Downregulated |
| Mki67 | Proliferation marker protein Ki-67 | Downregulated |
| Myh9 | Myosin-9 | Downregulated |
| Notch2 | Neurogenic locus notch homolog protein 2 | Downregulated |
| Pan3 | PAN2-PAN3 deadenylation complex subunit Pan3 | Downregulated |
| Parpbp | PARP1 Binding Protein | Downregulated |
| Prc1 | Protein regulator of cytokinesis 1 | Downregulated |
| Socs2 | Suppressor of cytokine signaling 2 | Downregulated |
| Spc24 | Kinetochore protein Spc24 | Downregulated |
| Spry2 | Protein sprouty homolog 2 | Downregulated |
| Srsf2 | Serine/arginine-rich splicing factor 2 | Downregulated |
| Stmn1 | Stathmin | Downregulated |
| Tet3 | Tet Methylcytosine Dioxygenase 3 | Downregulated |
| Tmsb10 | Thymosin | Downregulated |
| Top2a | DNA topoisomerase 2-alpha | Downregulated |
| Tpx2 | Targeting protein for Xklp2 | Downregulated |
| Ttk | Dual specificity protein kinase TTK | Downregulated |
| Uhrf1 | E3 ubiquitin-protein ligase UHRF1 | Downregulated |
| Wee1 | Wee1-like protein kinase | Downregulated |
