# Supplementary material for: Integrated Single-Cell Bioinformatics Analysis Reveals Intrinsic and Extrinsic Biological Characteristics of Hematopoietic Stem Cell Aging
Source: Front Genet. 2021 Oct 19;12:745786. doi: 10.3389/fgene.2021.745786 (PMC8560737; doi:10.3389/fgene.2021.745786)
Supplement: Supplementary file 4 [file Table3.DOCX]

| Supplementary Table 3: List for qPCR primers | |
| --- | --- |
| Gene | Sequence |
| Actb | GGCTGTATTCCCCTCCATCG |
|  | CCAGTTGGTAACAATGCCATGT |
| Aurka | CTGGATGCTGCAAACGGATAG |
|  | CGAAGGGAACAGTGGTCTTAACA |
| Aurkb | CAGAAGGAGAACGCCTACCC |
|  | GAGAGCAAGCGCAGATGTC |
| Casc5 | AGAGACACAGAAAACGCAGAC |
|  | ACATCCCACTCAGACAAACTCA |
| Ccna2 | GCCTTCACCATTCATGTGGAT |
|  | TTGCTGCGGGTAAAGAGACAG |
| Ccnb2 | GCCAAGAGCCATGTGACTATC |
|  | CAGAGCTGGTACTTTGGTGTTC |
| Cdca8 | CAAATTGAGTCCGACAGACAGA |
|  | GCCGAAGGATCTCGATGTTGT |
| Cdk1 | AGAAGGTACTTACGGTGTGGT |
|  | GAGAGATTTCCCGAATTGCAGT |
| Clec1a | ATGCAGGCCAAATACAGCAG |
|  | CCAGAATACAGGCTTATGGTGGT |
| Clu | AGCAGGAGGTCTCTGACAATG |
|  | GGCTTCCTCTAAACTGTTGAGC |
| Cxcr4 | GAAGTGGGGTCTGGAGACTAT |
|  | TTGCCGACTATGCCAGTCAAG |
| Egr3 | CCGGTGACCATGAGCAGTTT |
|  | TAATGGGCTACCGAGTCGCT |
| Ehd3 | AGTTGGCTGGGTAACGATGAT |
|  | GTGGTCTTGCCGGTAGAGT |
| Fignl1 | TGTGCAGGTGGACGAATGG |
|  | TGAACAGGTTGGTAGCAAAGAC |
| Gclc | CTACCACGCAGTCAAGGACC |
|  | CCTCCATTCAGTAACAACTGGAC |
| Gda | TCTGATGAGTGAACTTGGCAAC |
|  | CAATGTGCAATGGATGCTCCT |
| Gpr183 | ATGGCTAACAATTTCACTACCCC |
|  | CACCAGCCCAATGATGAAGAC |
| Gria3 | ACCATCAGCATAGGTGGACTT |
|  | ACGTGGTAGTTCAAATGGAAGG |
| Jun | CCTTCTACGACGATGCCCTC |
|  | GGTTCAAGGTCATGCTCTGTTT |
| Klhl4 | TATGGATGCTGCTAAAGGCAC |
|  | CAACAACTGCCACTCCAAATTG |
| Mt2 | GCCTGCAAATGCAAACAATGC |
|  | AGCTGCACTTGTCGGAAGC |
| Nupr1 | CCCTTCCCAGCAACCTCTAAA |
|  | TCTTGGTCCGACCTTTCCGA |
| Rorb | GCAGCATTAGCAATGGCCTC |
|  | GACGGCTGACCGGAATCTATG |
| Sbspon | CTGCTGCTTCGACTACGACAG |
|  | CCTCCGTTTAGTGGTTCCTGA |
| Sdpr | TCCTCTGATGACGAATTGCCC |
|  | GCTTTCTTGAGGCTATCGACTTT |
| Selp | CATCTGGTTCAGTGCTTTGATCT |
|  | ACCCGTGAGTTATTCCATGAGT |
| Slc14a1 | TTAAAGTAGACCGGGGTGAAAAC |
|  | GATTCCACTGATGGGGTTGC |
| Socs2 | GTGGGGAACTCGTCCTATCTG |
|  | GTCACAGTGTAATGATGTGCCA |
| Sult1a1 | CAACATGGAGCCCTTGCGTAA |
|  | ATGAGCACATCATCAGGCCAG |
| Tnfrsf1a | CCGGGAGAAGAGGGATAGCTT |
|  | TCGGACAGTCACTCACCAAGT |
| Ttk | GCAGTGTGACGATTGATTCCA |
|  | TCGGCACAGATTTTAGACAAGC |
