# Supplementary material for: Integrated Single-Cell Bioinformatics Analysis Reveals Intrinsic and Extrinsic Biological Characteristics of Hematopoietic Stem Cell Aging
Source: Front Genet. 2021 Oct 19;12:745786. doi: 10.3389/fgene.2021.745786 (PMC8560737; doi:10.3389/fgene.2021.745786)
Supplement: Supplementary file 5 [file Table4.DOCX]

Supplementary Method:

1. **Integrating datasets and clustering**

Three datasets of HSC expression profiles from young and aged mice (GSE100906, GSE70657 and GSE100426) were downloaded from the GEO database. To minimize batch effect between datasets, we integrated 3 datasets following the instructions of R package Seurat. Firstly, we performed standard preprocessing (log-normalization), and identified variable features individually for each dataset after quality control. Functions NormalizeData and FindVariableFeatures were used to identify the top highly variable 2000 genes and the selection method was set to “vst”. Next, we identified anchors using the FindIntegrationAnchors function (dims = 1:30) and passed these anchors to the IntegrateData function (dims = 1:30), which returned a Seurat object with integrated data. This new integrated matrix was used for downstream analysis and clustering. Then the ScaleData function of Seurat was used to calculate the scaling expression values and the RunPCA function (npcs = 30) was used to perform a PCA dimensionality reduction by Uniform Manifold Approximation and Projection (UMAP). To cluster the cells, we next applied the FindNeighbors and FindClusters Seurat functions (resolution = 0.8). Function FindAllMarkers was used to identify markers that define clusters via differential expression (only.pos =TRUE, min.pct = 0.25). Visualization of the results was based on the Seurat functions, including DimPlot, DoHeatmap and VlnPlot.

1. **Identification of DEGS between young and aged HSCs**

Firstly, we applied some quality control threshold to remove cells that may not have good quality. Unique feature counts over 6,000 or less than 200 were removed and we calculated mitochondrial QC metrics with the PercentageFeatureSet function and filtered cells that have >10% mitochondrial counts. After removing unwanted cells from the dataset, we employed a global-scaling normalization method “LogNormalize” in function NormalizeData (scale factor 10,000 by default) to normalize the data. We next calculated a subset of features that exhibit high cell-to-cell variation in the dataset by using function FindVariableFeatures. After scaling the data, we identified the DEGs by functions FindAllMarkers (return.thresh = 0.05).

1. **Clustering-based analysis of cell cycle state**

The cell cycle phase (G1, S, or G2M) of each cell was identified by calculating the cell cycle phase score based on canonical markers using the Seurat package. Function CellCycleScoring was used with the default parameters. The canonical cell cycle gene set was defined with “cell cycle process” from the GO annotation and identified as cycling in HeLa cells and it can be downloaded from GSEA MSigDB version 7.1 (https://www.gsea-msigdb.org/gsea/msigdb).

1. **Outgoing communication patterns analysis of interactions between HSCs and surrounding cells in the bone marrow niche**

To identify which cell population accounted for the difference in inflammatory cytokine levels, CellChat software was applied to analyze outgoing communication patterns of secreting cells. Firstly, two CellChat objects with metadata (young and aged bone marrow dataset) were created by function createCellChat. CellChatDB mouse database was used to predict the cell-cell contact interactions. Secondly, identifyOverExpressedGenes (thresh.p = 0.05) and identifyOverExpressedInteractions Cellchat function were used to identify over-expressed ligands or receptors and over-expressed ligand-receptor interactions. Then computeCommunProb function (methods for computing the average gene expression per cell group was set as "triMean") was used to infer the biologically significant cell-cell communication. NetAnalysis_contribution function was used to compute the contribution of each ligand-receptor pair to the overall signaling pathway (threshold of the p-value=0.05). The receptors expressed by HSPCs and the ligands expressed by surrounding cells in the bone marrow niche were considered in the downstream analysis. Visualization signaling pathways were realized by functions netAnalysis_contribution and netVisual_aggregate.
